# Pyo-Salpinx in Its Relation to Puerperal Fever

**Published:** 1887-06

**Authors:** 


### Selections.

### *PYO-SALPINX IN ITS RELATION TO PUERPERAL FEVER.*

The following report, taken from the transactions of the Philadelphia Obstetrical Society, is full of interest, because it shows a possible etiological factor in the causation of puerperal fever heretofore taken into consideration too infrequently. Because of its exceeding great practical interest, we give the synopsis in full.

Dr. J. M. Baldy presented this specimen, not simply because it was one of pyo-salpinx, but because of its extremely important relation to the puerperal state, and, as far as he is aware, because it is the first of its kind ever operated upon, and life saved when the patient was dying from so-called puerperal fever.

The patient, Mamie P., 23 years of age, was delivered of a male child after a tedious but normal labor some four years ago. She was at that time confined to her bed for eight weeks with "an inflammation in her stomach." She, however, made a good recovery, and has not suffered with pain or ache in her abdomen since. On February 3, 1887, he was called to attend her in her second labor. Although he went with the messenger, he found labor over; a dead child, together with the placenta and all the membranes intact, lay between her thighs. Her uncovered arms, chest and legs were exposed in a room without a fire. No examination was made, but she was put between warm, dry bed-clothes as quickly as possible. On the second or third day she had a chill with a quick rise of pulse and temperature, a tympanitic and tender abdomen. These symptoms abated somewhat, and he lost sight of her several weeks. On the third of March, one month after her confinement, he was again summoned to her, and found that she had been suffering ever since he had seen her. She had become so emaciated that he hardly recognized her. Her temperature was 102°, and pulse 130. She had continued chills and creeps, hectic, night-sweats and sleepless nights; her abdomen was swollen and tympanitic, and intensely painful, her bowels loose and fœtid; micturition and defecation were both painful. She was evidently fast approaching death. An examination of the soft parts showed no sign of a recent tear. The uterus was subinvoluted, and on the left side there was a large boggy mass firmly adherent, tortuous, and extremely tender. The right side was tender, but no mass could be detected. Abdominal section was advised as the only hope of saving her life, and the proposition was eagerly accepted by the patient and her friends. Dr. J. Price saw the patient, and confirmed this opinion of immediate operation. He operated on March 5th, the delay being necessary in order to allow the surroundings being cleansed. Drs. J. Price, McMurtrie, of Danville, Ky., and Mr. Eckman, of Scranton, Pa., were assisting. The right tube and ovary were healthy, and were not removed; left tube was almost as large as the uterus, and firmly adherent in all directions, especially to the bowels, from which it was separated with great difficulty. An abscess of the cellular tissue was ruptured while breaking up the adhesion, and pus welled up through the abdominal incision. Both tube and ovary were removed. A large cheesy mass on the bowel at the point of adhesion was trimmed down with scissors, and Monsel's solution applied to the bleeding points. After a free irrigation, a drainage tube was put in, and the incision, which was only one and a-half inches in length, was closed. The tube was found to be distended with pus, the ovary was disintegrated, and contained pus. The patient rallied quickly and had no shock, her pulse fell 80, and her temperature to normal within twelve hours, and remained so. The tube was removed on the seventh day. There had been little or no pain; no catheter, no laxative or drug of any kind had been employed. The day after the removal of the tube her pulse began to rise, as also did her temperature. Pain developed in the left ovarian region, and she began to have hectic and creeps. About the eleventh day there was a free gush of pus from the tube tract, and she began to improve again from that moment. A rubber tube was inserted and passed deep into the pelvis, and the abscess was washed out twice daily. The discharge gradually diminished, and the tube was again removed. The wound is now completely healed, and the patient is a well woman.

The belief that a certain proportion of our puerperal fever cases are simply cases of salpingitis septicus is by no means a new one, and is probably held by most of the great operators in the world. Dr. M. Sänger says that "salpingitis septicus co-existing with severe puerperal septicæmia has never as yet given the surgeon an opportunity to remove the principal focus of disease by the extirpation of the tubes. It is possible, however, that under certain circumstances such a procedure might be indicated." Dr. Carl Schroeder holds that "septic endometritis does not extend to the tubes as a rule; occasionally, however, it does go on to a purulent salpingitis." That these cases do exist much more frequently than we have had any idea of is certain, and that oftentimes a life, otherwise doomed, can be saved by operative interference is proved by the case presented to-night. Mr. Tait mentions four deaths from this cause in Queen Charlotte Hospital alone, and says "that these cases were, during life, all regarded as puerperal fever." Dr. A. Martin, out of a total of two hundred and eighty-seven cases, found that seventy resulted from the puerperal state. Dr. Sänger mentions two cases which have come to knowledge in which the over-distended tubes burst and discharged pus into the abdominal cavity with death on the fourth day after confinement in one case, and on the twenty-first day in the second case. He thinks that in both these cases the salpingitis existed before delivery, and mentions a case in his own practice in which this certainly was the condition. Hecker, as early as 1878, mentions two cases in which the pyo-salpinx was old and was only lit up by the puerperal state. Whether the disease arises *de novo*, or having already existed from other causes is simply lit up by the puerperal state, must be determined in each individual case. Hecker's and Sänger's cases as mentioned had a pre-existing salpingitis, but in the seventy cases reported by Martin, the micro-organisms of puerperal septicæmia were found in the contents of the tubes, and no mention is made of any other micro-organism; so it is fair to presume that these cases arose from the puerperal state pure and simple. Of course, the possible contagion of gonorrhœa can never be eliminated except by a microscopic examination. In his case, although the trouble seemed very clearly to have arisen at the second time of labor, possibly with her first labor also, yet the chance of gonorrhœal infection, both before and after her first pregnancy, are so great that he cannot pretend to say it was not present. The operation has up to this time been done at least four times in Philadelphia; one case was operated on just two weeks previous to mine by Dr. Longaker, in which a pyo-salpinx was found and removed, the patient dying on the second day. Dr. J. Price has since operated twice, and in one found more than a quart of pus in the abdominal cavity. The case, unfortunately, fell into his hands too late, and the patient only survived two days. These cases, though few in number, certainly teach us that the work done in this direction is encouraging, and although a large percentage have died, it only warns us of the extreme importance of an early diagnosis and prompt surgical interference. It becomes our imperative duty in every case of post puerperal trouble to make a thorough investigation of the appearance of the first symptoms, and should a fullness be found on either or both sides of the uterus, accompanied by pain on touch, and with constitutional symptoms of gravity, there should be no hesitation as to the course to pursue. This being secured, our present high mortality of one woman out of every hundred deliveries in large cities, as recently stated in a statistical paper on lying-in charities in the United States, must be largely diminished, and the fatal influences now surrounding our purulent women must become infinitely less.

In the discussion which followed, Dr. Tait read the following letter from Mr. Lawson Tait:

"There can be no doubt as to the frequency of the occurrence of puerperal pyo-salpinx; and what we want to do is to hammer at people until we get them to open the abdomen in primary puerperal peritonitis."

